# Whole genome sequencing of multidrug resistant Enterobacterales identified in children and their household members within Siem Reap, Cambodia

**DOI:** 10.1093/jacamr/dlad067

**Published:** 2023-06-14

**Authors:** Shweta R Singh, Cheng Yee Tang, Bunsoth Mao, Sona Soeng, Clare L Ling, Jocelyn Qi-Min Teo, Saphonn Vonthanak, Paul Turner, Li Yang Hsu, Rick Twee-Hee Ong

**Affiliations:** Saw Swee Hock School of Public Health, Tahir Foundation Building, National University of Singapore, Singapore; Saw Swee Hock School of Public Health, Tahir Foundation Building, National University of Singapore, Singapore; University of Health Sciences, Phnom Penh, Cambodia; Cambodia Oxford Medical Research Unit, Angkor Hospital for Children, Siem Reap, Cambodia; Cambodia Oxford Medical Research Unit, Angkor Hospital for Children, Siem Reap, Cambodia; Centre for Tropical Medicine and Global Health, Nuffield Department of Clinical Medicine, University of Oxford, Oxford, UK; Department of Pharmacy, Singapore General Hospital, Singapore; University of Health Sciences, Phnom Penh, Cambodia; Cambodia Oxford Medical Research Unit, Angkor Hospital for Children, Siem Reap, Cambodia; Centre for Tropical Medicine and Global Health, Nuffield Department of Clinical Medicine, University of Oxford, Oxford, UK; Saw Swee Hock School of Public Health, Tahir Foundation Building, National University of Singapore, Singapore; Department of Medicine, Yong Loo Lin School of Medicine, NUHS Tower Block, National University of Singapore, Singapore; Singapore Centre on Environmental Life Sciences Engineering (SCELSE), Nanyang Technological University, Singapore; Saw Swee Hock School of Public Health, Tahir Foundation Building, National University of Singapore, Singapore

## Abstract

**Objectives:**

To explore the association of recent hospitalization and asymptomatic carriage of multidrug-resistant Enterobacterales (MDRE) and determine the prevailing strains and antibiotic resistance genes in Siem Reap, Cambodia using WGS.

**Methods:**

In this cross-sectional study, faecal samples were collected from two arms: a hospital-associated arm consisted of recently hospitalized children (2–14 years), with their family members; and a community-associated arm comprising children in the matching age group and their family members with no recent hospitalization. Forty-two families in each study arm were recruited, with 376 enrolled participants (169 adults and 207 children) and 290 stool specimens collected from participants. The DNA of ESBL- and carbapenemase-producing Enterobacterales cultured from the faecal samples was subject to WGS on the Illumina NovaSeq platform.

**Results:**

Of the 290 stool specimens, 277 *Escherichia coli* isolates and 130 *Klebsiella* spp. were identified on CHROMagar ESBL and KPC plates. The DNA of 276 *E. coli* (one isolate failed quality control test), 89 *Klebsiella pneumoniae*, 40 *Klebsiella quasipneumoniae* and 1 *Klebsiella variicola* was sequenced. CTX-M-15 was the most common ESBL gene found in *E. coli* (*n* = 104, 38%), *K. pneumoniae* (*n* = 50, 56%) and *K. quasipneumoniae* (*n* = 16, 40%). The prevalence of bacterial lineages and ESBL genes was not associated with any specific arm.

**Conclusions:**

Our results demonstrate that MDRE is likely to be endemic within the Siem Reap community. ESBL genes, specifically *bla*_CTX-M_, can be found in almost all *E. coli* commensals, indicating that these genes are continuously propagated in the community through various unknown channels at present.

## Introduction

The increasing incidence of MDR in bacterial pathogens has emerged as a silent epidemic. MDR pathogens reduce antimicrobial efficacy causing prolonged illness and increasing the burden on strained healthcare systems.^1,2^ MDR Enterobacterales (MDRE), including ESBL-producing Enterobacterales (ESBL-E) and carbapenem-resistant Enterobacterales (CRE), are identified as ‘critical priority’ resistant organisms by the WHO.^3^ Initially associated with clinical infections acquired within healthcare institutions, lately ESBL-E have increasingly been found in community-acquired infections.^4^

*Klebsiella pneumoniae* and *Escherichia coli* are two important commensal organisms found in the human gut and are also common causes of extra-intestinal infections. Carriage of ESBL-E has been identified as a risk factor for clinical infection.^5,6^ Recent reviews on faecal ESBL-E carriage have shown an increasing trend in the ESBL-E carriage rates in healthy individuals worldwide and specifically in the Southeast Asian region.^7,8^ To control MDRE spread, it is essential to identify the reservoirs and dissemination paths of resistant bacteria and antibiotic resistance genes (ARGs) that have contributed to this public health crisis.

In Cambodia, several studies have been conducted to investigate the incidence of MDRE; a study conducted on clinical specimens reported increasing proportions of ESBL-E infections, from 24% in 2012% to 38% in 2015.^9^ Another study in the same hospital’s outpatient department showed that 55% of children were colonized by extended-spectrum cephalosporin-resistant Enterobacterales (*E. coli* and *K. pneumoniae*).^10^ Risk factors associated with colonization included recent hospitalization and presence of intestinal parasites. However, in a recent cross-sectional study conducted in 2019, there was no difference in ESBL-E colonization prevalence in recently hospitalized children and their families compared with children and individuals with no recent history of hospitalization. The prevalence of ESBL-E colonization (92% ESBL *E. coli* and 44% *K. pneumoniae*) reported in the study was higher compared with all previous studies in Cambodia.^11^ The aim of this study was therefore to determine the population structures of the bacterial strains and ARGs present in *E. coli* and *K. pneumoniae*, particularly of ESBL genes from the 2019 study, which could identify potential reservoirs of ARGs.

## Methods

### Patients and setting

Faecal samples were collected from community participants of Siem Reap, Cambodia in a cross-sectional study from August to November 2019. The study included two arms: a hospital-associated household (where a child aged 2–14 years was hospitalized in the past 14–28 days for a minimum 48 h); and a community-associated household, where a child in a matching age group (2–14 years) and other family members were not hospitalized in the past 12 months. The children in the hospital-associated arm were identified and recruited from Angkor Hospital for Children (AHC) in Siem Reap and approached between 14 and 28 days after discharge. Data collectors interviewed consenting residents who had lived in the household for at least 1 month before the recruitment day, using a tablet-based questionnaire on the Qualtrics platform. We obtained parental consent for participants under 18 years old and separate assent for children aged 7 years and above. We made repeated visits to each household to recruit additional household members if needed, and there were no exclusion criteria for other residents in the households.

Households were approached in the same street or village as the hospital-associated households that had a child of similar age (±2 years) to the index child. On the same day, households where the age-matched child and any other family member had not been hospitalized in the past year were eligible for inclusion in the study as community-associated households. We recruited the first eligible community household that agreed to participate in the study.

### Microbiological methods

The 290 faecal samples were stored at −80°C in tryptone soya broth/10% glycerol medium immediately on receipt at the laboratory and thawed for interval processing in batches. The samples were cultured to detect ESBL-producing *E. coli* and *K. pneumoniae* using chromogenic media (ESBL, KPC agar; CHROMagar, Paris, France; prepared in-house). Suspect colonies (one distinct colony per sample) were confirmed with MALDI-TOF (VITEK MS, database V3.2; bioMérieux, Marcy L’ Etoile, France). Antimicrobial susceptibilities were examined by disc diffusion, and zone diameters were interpreted using the CLSI guidelines, 2019 version.^12^ ESBL and carbapenemase production were confirmed using double-disc diffusion and the modified carbapenem inactivation method (mCIM), respectively. Non-susceptibility was defined as isolates resistant or intermediate susceptible to antimicrobials. The subcultured isolates (ESBL and carbapenemase-positive *E. coli* and *K. pneumoniae*) were stored frozen at −80°C in skim milk/tryptone/glucose/glycerol medium.

### WGS

DNA was extracted from subcultured isolates using the Promega Wizard Genomic DNA purification kit, following the manufacturer’s instructions. The DNA samples were then tested for purity and integrity using agarose gel electrophoresis followed by Qubit 3.0 fluorometer quantitation. Samples with a DNA yield greater than 400 ng were library prepped using the NEBNext^®^ Ultra^™^ II DNA Library Prep Kit. Post further quality checks and purification, pooled DNA libraries were sequenced on the Illumina^®^ NovaSeq 6000 sequencing platform to yield 150 bp paired-end reads.

### Bioinformatics analysis

The raw sequence reads were trimmed to remove adaptors and low-quality bases using Trimmomatic (v0.32),^13^ and were subsequently de novo assembled using SPAdes (v3.11.1).^14^ Species identification was performed using Mash Screen (v2.3) by comparing the raw sequencing reads with a set of species database curated by the Kleborate (downloaded on 23 June 2021).^15^ All samples were subjected to *in silico* MLST against the PubMLST database (https://pubmlst.org) (downloaded on 31 August 2021) using a custom Python script integrating BLASTn. The Achtman MLST scheme was used for *E. coli*, whereas the Pasteur MLST scheme was used for *K. pneumoniae*, *K. quasipneumoniae* and *K. variicola*. The *E. coli* isolates were classified in phylogenetic groups based on the presence/absence of genes *chuaA*−, TspE4.C2− (Group A); *chuA*−, *yjaA*−, TspE4.C2+ (Group B1); *chuA*+, *yjaA*+ (Group B2); and *chuA*+, *yjaA*− (Group D).^16^ The presence of ARGs in the assembled nucleotide sequences was identified using AMRFinderPlus (v3.10.1) with its own database (version 2021-03-01.1) using the gene sequence coverage and identity threshold set as >80% and >90%, respectively.^17^ All genome assemblies were subjected to additional genotyping using Kleborate (v2.0.4).^18,19^ Plasmid incompatibility groups were identified using PlasmidFinder (v2.0.1) with its own database (version 2021-07-12).^20^ The genetic context of specific *bla*_CTX-M_ and *bla*_NDM-5_ genes was examined by extracting the contigs containing these genes and annotating the contigs using Prokka (v1.14.6).^21^ ISs in the contigs were verified using IS finder.^22^ The contigs that contained the same *bla*_CTX-M_ allele variant were clustered using CD-HIT (v4.8.1)^23^ and verified using BLASTn. Schematics of the gene features were visualized using R (v4.1.0) ggplot2^24^ and gggenes packages.^25^

Comparison of genes between the two arms (hospital and community) for the presence or absence of certain genes was tested using chi-square test. Fisher’s exact test was used when the sample size of prevalent genes was below 30.

For phylogenetic analysis, trimmed reads were aligned to an appropriate reference genome: AE014075.1 *E. coli* CGT073 strain or CP000647.1 *K. pneumoniae* subsp. *pneumoniae* MGH 78578 strain. The core genome alignments were generated using Snippy (v4.6.0).^21^ Recombination sites were removed using Gubbins (allowing 28% missing data threshold for *E. coli* and 25% missing data threshold for *K. pneumoniae*).^26^ Pairwise SNP distance was calculated from the recombination-free core genome alignment using snp-dists.^27^ Maximum likelihood trees were constructed from the recombination-free core genome alignment with IQ-TREE (v2.0.3)^28^ and visualized using R ggplot2 and ggtree packages.^29^

## Results

**Figure 1.**
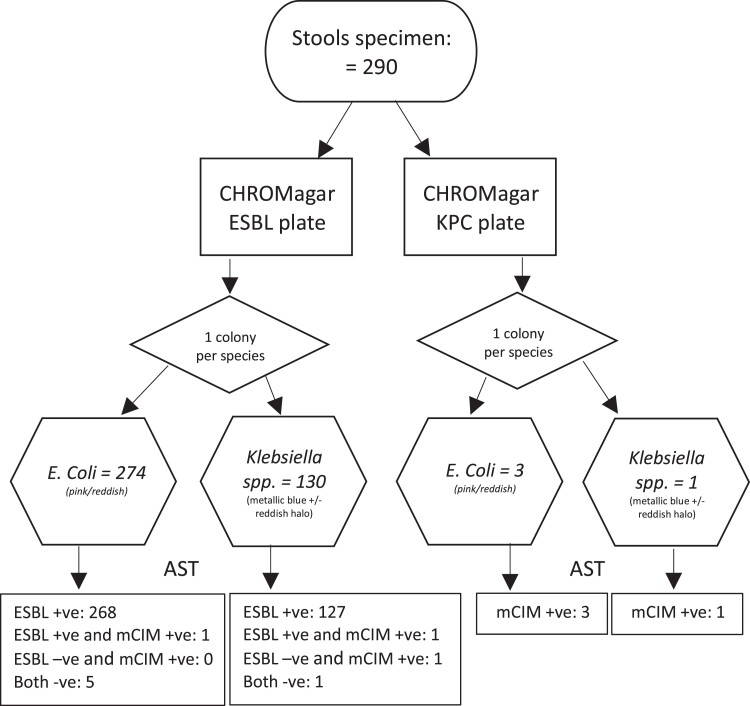
Phenotype detection of ESBL *E. coli* and *Klebsiella* spp. isolates. mCIM, modified carbapenem inactivation method.

Stool samples were collected from 290 participants in 84 families (42 family units in each arm). A total of 277 *E. coli* were identified on the CHROMagar plates of which 269 (92.8%, 269/290) were ESBL positive and 5 isolates were ESBL negative but third-generation cephalosporin-resistant. Four *E. coli* isolates were positive for carbapenemase, of which one was also positive for ESBL. *K. pneumoniae* was isolated from 131 ESBL and KPC CHROMagar plates, of which 128 (44.1%, 128/290) were confirmed as ESBL-positive and 3 as mCIM-positive. Two of the three mCIM-positive *K. pneumoniae* isolates came from the same individual (cultured from different CHROMagar plates) and hence only one isolate was used for further processing. All of the 407 (277 *E. coli* and 130 *K. pneumoniae*) identified isolates had their DNA extracted and submitted for WGS (Figure 1). Of these, the DNA of one *E. coli* isolate failed to meet the DNA quality threshold and therefore sequence data were available for 406 isolates. The fastq reads for the 406 isolates have been uploaded to NCBI, and the project assession number is PRJNA885285.

Geontypic species identification from the sequence data confirmed all 276 phenotypic *E. coli* samples as *E. coli*/Shigella (273) and *Escherichia* cryptic clade-1 (3). For the 130 phenotypic *K. pneumoniae* isolates, Kleborate identified 89 to be *K. pneumoniae*, 40 as *K. quasipneumoniae* and 1 as *K. variicola*. The prevalent *E. coli* STs were ST10 (*n* = 17), ST131 (*n* = 15), ST1193 (*n* = 14) and ST38 (*n* = 13), as shown in Table S1 (available as Supplementary data at *JAC-AMR* Online). The major STs for *K. pneumoniae* isolates were ST101 (*n *= 9), ST37 (*n* = 4), ST1741 (*n* = 4) and ST967 (*n* = 4) whereas for *K. quasipneumoniae* the major STs were ST1308 (*n* = 8), ST841 (*n* = 4) and ST1584 (*n* = 4), as shown in Table S2. There were no differences in proportions of the STs for any of the ESBL-E (*E. coli*, *K. pneumoniae* and *K. quasipneumoniae*) between the hospital-associated households and community-associated households.

The gene *bla*_CTX-M-15_ was the most commonly found ESBL gene in *E. coli* (*n* = 104, 38%), *K. pneumoniae* (*n* = 50, 56%) and *K. quasipneumoniae* (*n* = 16, 40%). The second most common ESBL gene in *E. coli* was *bla*_CTX-M-55_ (*n* = 90/32%), followed by *bla*_CTX-M-27_ (*n* = 48/17%). The *bla*_CMY-42_ gene was found in all five *E. coli* ESBL-negative third-generation cephalosporin-resistant isolates, whereas the *bla*_SHV_ gene and its variants were present in one ESBL-negative and four phenotypically ESBL-positive *K. pneumoniae*. The distribution of the ESBL genes across the three species is summarized in Tables 1 and 2. CTX-M-27 was most commonly found in *E. coli* with ST131 and ST1193, although the association was not statistically significant (Table S3). *K. pneumoniae* and *K. quasipneumoniae* distribution of major ST and ESBL genes is given in Table S4.

**Table 1.** Distribution of ESBL *E. coli* (*n = *276) phenotypes and major genes

Genes	Hospital arm	Community arm	Total	*P* value^a^
CTX-M-15	57	47	104	0.37
CTX-M-27	33	17	50	0.32
CTX-M-14	13	8	21	0.93
CTX-M-55	52	38	90	0.93
CTX-M-9	—	—	—	
CTX-M-3	0	1	1	
CTX-M-24	2	1	3	
CTX-M-65	1	2	3	
CTX-M-63	—	—	—	
*bla*_CMY-2_-positive^b^	6	0	6	
*bla*_SHV_-positive				
SHV-2/2-like	—	—	—	
SHV-12	—	—	—	
SHV-26^c^ + 238S	—	—	—	
Carbapenemase-positive				
*bla*_NDM-5_-positive	4	0	4	
*bla*_OXA-48_-positive	1	0	1	

a Chi-square/Fisher’s exact test.

b Three *bla*_CMY-2_-positive *E. coli* also possessed CTM-M-15 and CTX-M-27 genes.

c Three *E. coli* isolates possessed more than two types of ESBL acquired genes.

**Table 2.** ESBL-*K. pneumonia* and ESBL-*K. quasipneumoniae* phenotypes and major genes

Genes	*K. pneumoniae* (*n* = 90)				*K. quasipneumoniae* (*n* = 40)			
	H	C	Total	*P* value^b^	H	C	Total	*P* value^a^
CTX-M-15	29	21	50	0.86	11	5	16	0.41
CTX-M-27	3	2	5		7	3	10	0.44
CTX-M-14	3	3	6		2	3	5*	
CTX-M-55	5	2	7		2	1	3	
CTX-M-9	1	4	5		1	1	2	
CTX-M-3^b^	2	2	4		0	1	1	
CTX-M-24	0	2	2		—	—	—	
CTX-M-65	0	0	0		—	—	—	
CTX-M-63	1	0	1		—	—	—	
SHV-2/2-like	1	2	3		0	2	2	
SHV-12	—	—	—		0	2	2	
SHV-26* + 238S	1	0	1		—	—	—	
Carbapenemase-positive								
*bla*_NDM-5_-positive	2	0	2		—	—	—	
*bla*_OXA-48_-positive	—	—	—		—	—	—	

H, Hospital arm; C, Community arm.

a Chi-square/Fisher’s exact test.

b The one *K. variicola* isolate had the CTX-M-3 ESBL gene.

The *bla*_NDM-5_ gene was present in all of the six carbapenemase-producing (CP) Enterobacterales (four *E. coli* and two *K. pneumoniae*). The STs for CP *E. coli* isolates were ST167, ST410 (*n* = 2) and ST648. One *E. coli* ST131 isolate was identified to harbour *bla*_OXA-48_ from the sequence data, which is not surprising given that CHROMagar KPC plates are not 100% sensitive or specific.^30^ The STs of the two CP *K. pneumoniae* isolates were ST45 and ST11.

### Likely transmissions within the community

**Figure 2.**
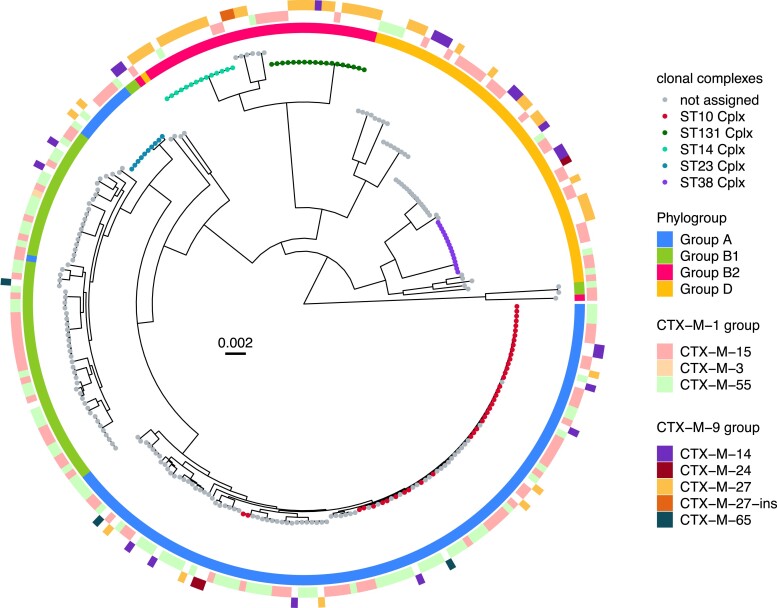
Recombination-filtered core SNP maximum likelihood phylogenetic tree for 277 *E. coli* isolates. The tree was midpoint-rooted. The tips were coloured by the ST clonal complexes. Inner circle shows the phylogroup of the isolates; middle circle shows the presence of CTX-M-1 group genes identified in the isolates; outer circle shows the presence of CTX-M-9 group genes identified in the isolates. Scale bar indicates the number of nucleotide substitutions per site.

**Figure 3.**
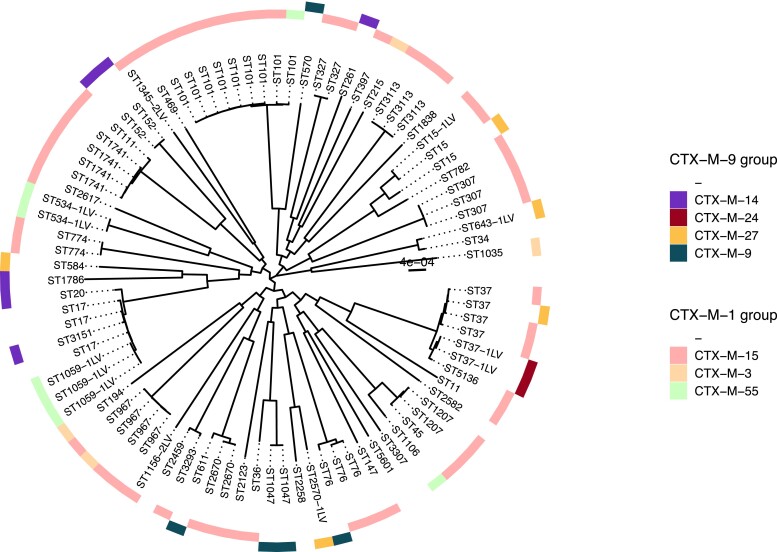
SNP phylogenetic circular tree for 131 *K. pneumoniae* isolates. The tree was midpoint-rooted. The tips were coloured by the ST clonal complexes. Inner circle shows the phylogroup of the isolates; middle circle shows the presence of CTX-M-1 group genes identified in the isolates; outer circle shows the presence of CTX-M-9 group genes identified in the isolates. Scale bar indicates the number of nucleotide substitutions per site.

To identify potential transmissions, we assessed the core phylogenies of the *E. coli* and *K. pneumoniae* isolates as illustrated in Figures 2 and 3. Using a threshold of 10 SNPs, we identified: (i) 18 instances of potential transmission for *E. coli* of which 10 instances were from individuals in nine families (Table S5); and (ii) 11 instances of potential transmission for *K. pneumoniae* in seven families (Table S6). In the 18 instances of potential transmission for *E. coli*, the majority consisted of the combination of ST131/1193 with CTX-M-27 (three instances) found both within and across different households. This was followed by ST10 and ST215 with the ESBL gene CTX-M-15 (Table 3). With respect to *K. pneumoniae,* ST101 with CTX-M-15 was found in two separate possible transmission instances in individuals belonging to different households.

**Table 3.** Distribution of prevalent AMR genes present in non-susceptible *E. coli* and *K. pneumoniae*

Antibiotics	*E. coli* (*n* = 276)		Total (%)	*K. pneumoniae* (*n* = 89)		
	Total (%)	Common genes		Total (%)	Common genes	Total (%)
Colistin	5 (1.8)	mcr3.1	4 (80)	—	—	
		mcr1.1	2 (40)			
Ciprofloxacin	252 (91.3)	qnrS1/S1*	132 (52.4)	83 (93.3)	qnrS1/S1*	58 (69.9)
Co-trimoxazole/sulfamethoxazole/trimethoprim	191 (69.2)	sul2 and/or sul1	152 (79.6)	60 (67.4)	sul2/S*	46 (76.7)
		dfrA17	74 (38.7)		dfrA14.v2*	25 (41.7)
Chloramphenicol	87 (31.5)	floR.v1/v1*	22 (25.3)	23 (25.8)	floR.v1	11 (47.8)
		catII.2*	17 (19.5)			
		catA1*/A1^	16 (18.4)			
Gentamicin	82 (29.7)	aac(3)-iid^	45 (54.9)	19 (21.3)	aac(3)-iid^	11 (57.9)

### Presence of other ARGs besides ESBL

WGS also enabled the identification of genes that are associated with resistance to several antibiotics including ciprofloxacin, co-trimoxazole, chloramphenicol and gentamicin (Table 3). Ciprofloxacin non-susceptible (NS) *E. coli* and *K. pneumoniae* isolates most commonly possessed the genes qnrS1/S1*. *E. coli* isolates non-susceptible to co-trimoxazole were found to harbour the sulfamethoxazole resistance gene sul2/2*/2^ (*n* = 52, 27.22%), sul1/1* (*n* = 40, 21%) and their combination *sul1*;*sul2* (*n* = 60, 31.4%). The gene s*ul2* was also most prevalent in NS *K. pneumoniae* (*n* = 46, 76.7%) followed by *Sul1* (*n* = 35, 58.3%). However, a number of NS isolates (nine *E. coli* and three *K. pneumoniae*) were not found to harbour any sul- or tmt (trimethoprim)-specific genes, suggesting that there might be other mechanisms for resistance. Colistin-resistant genes *mcr3.1* (*n* = 4) and *mcr1.1* (*n* = 2) were found in five *E. coli* isolates (one isolate possessed both colistin resistance genes). The phenotypic susceptibilities to major classes of antibiotics—ciprofloxacin, co-trimoxazole, chloramphenicol and gentamicin—and their corresponding genes (identified using Kleborate) are presented in Tables S7-S9.

### Genetic context of bla_CTX-M-27_

The *E. coli* isolates possessing ESBL *bla*_CTX-M-27_ genes were further analysed for their genetic context. Of these 50 isolates, 18 (36%) were likely to be on plasmids and 4 (8%) were likely of chromosomal origin, whereas locales could not be adequately determined for the remaining isolates (Figure S1). The CTX-M-27-containing contigs are unfortunately not typeable, whereas the remaining contigs predicted to be from plasmids (lengths ranging from 1470 to 6590 bp) had similar sequence identities (>90% similarity and 100% query coverage) to several plasmid sequences such as CP054458.1 and CP088684.1. All of the E.coli *bla*_CTX-M-27_ genes were found to harbour IS*Ecp1* upstream, with lengths ranging from 387 to 540 bp in 30 isolates and truncated in the remaining 10 isolates. IS*903B* was consistently found downstream, with lengths ranging from 388 to1057 bp in 37 isolates (only three did not have the IS*903B*). The *K. pneumoniae* and *K. quasipneumoniae* isolates with *bla*_CTX-M-27_ also showed similar genetic structure with IS*Ecp1* upstream and IS*903B* downstream, with lengths of 1656 and 598 bp or unknown respectively (Figure S2). For *K. pneumonia*e, the plasmid replicon IncFIA(HI1) was identified to be present within a CTX-M-27-containing contig only.

### Genetic context of bla_NDM-5_ in isolates from same individual

**Figure 4.**
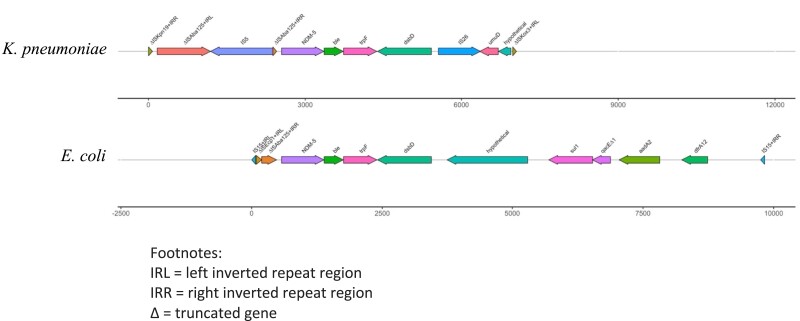
Schematic of the genetic structure of the contigs harbouring NDM-5 in *K. pneumoniae* and *E. coli* isolated from an individual.

The genetic structure of *bla*_NDM-5_ found in *E. coli* and *K. pneumoniae* from the same individual (an infant with no medical or hospitalization history in the past 1 year) was also analysed. The contig length harbouring *bla*_NDM-5_ in the *E. coli* isolate differed (9827 bp) substantially from the *K. pneumoniae* isolate (7054 bp), where only 49% of the *K. pneumoniae* and 35% of the *E. coli bla*_NDM-5_ contigs matched, indicating that the genetic environment was not similar between *E. coli* and *K. pneumoniae* (Figure 4). The assembled contigs were, however, identical within the same species isolated from different individuals.

## Discussion

The WGS and subsequent analysis showed that there was no significant difference in the ESBL genes and MDRE strains depending on the arm, and thus recent hospitalization has no association with a specific ESBL gene or strain. The significant prevalence and substantial genetic diversity of the ESBL genes within Siem Reap highlight that ESBL-producing *E. coli* and *K. pneumoniae* are likely to be endemic in the community. The results from this study also indicate that there are no major differences in the stool specimen-cultured *E. coli* and *K. pneumoniae* genotypes in families of recently hospitalized children compared with families with children that had no recent hospitalization. Our study contrasts with findings from another study from Spain where the index patient and their household members had higher prevalence of ESBL-E compared with non-hospitalized community individuals.^31^ The bacterial strains and ESBL genes were also very diverse in our sample, with several instances of possible transmission identified. The possible transmissions were, however, distinct across the clusters harbouring different sequence types and ESBL genotypes. There were several transmission clusters with known *E. coli* strains such as ST131 and ST1193 that had been identified as ‘high-risk clones’, spreading across all regions in the world.^32–34^ In two separate transmission clusters, *K. pneumoniae* ST101 was identified, hinting at a common environmental link of possible transmission. Previous studies have linked a *K. pneumoniae* ST101 clone with not only harbouring carbapenemase resistance but also persisting in the environment through hospital wastewater effluent.^35,36^ The high pervasiveness and isolation from unrelated individuals warrants further inspection of environmental sources that could be disseminating the ‘high-risk clones’ in the community.

The predominant ESBL gene family in our isolates was *bla*_CTX-M_, with major allelic variants being *bla*_CTX-M-15_, *bla*_CTX-M-55_ and *bla*_CTX-M-27_, which are also commonly found elsewhere in the Greater Mekong region.^8,9^ We also identified carbapenem resistance in four *E. coli* and two *K. pneumoniae* isolates, all of which possessed the *bla*_NDM-5_ gene. In this study, all but one individual with NDM-5-positive isolates had no history of a recent hospitalization. We also identified a carbapenem (meropenem)-susceptible *E. coli* isolate that possessed the *bla*_OXA-48_ gene on sequencing. Although CHROMagar KPC plates are not 100% sensitive, especially to OXA-48-like carbapenemase, it is surprising that the *E. coli* strain possessing OXA-48-like carbapenemase was susceptible to meropenem, implying that the OXA-48 gene is not expressed phenotypically.^30^ The species-specific *bla*_NDM-5_ contigs were identical thus hinting at potential transmission from a common source, particularly because all the positive isolates came from individuals in different households. However, we could not identify a common source of transmission or a link between the individuals as they belonged to different villages and had no history of recent hospitalization. Given the limited number of positive samples, we were not able to identify any independent risk factors associated with colonization by a *bla*_NDM-5_-containing strain. With respect to *K. pneumoniae,* we did not identify high-risk clones of *K. pneumoniae* such as ST15^37^ and ST147^38^ in our sample population. However, it was alarming to identify high-risk clones of ST101^36^ and ST11^39^ in the community, especially ST101, which was also found in possible transmission events from two separate instances. ST101 is especially known as a ‘superbug’ that carries both virulence and a combination of carbapenem and colistin resistance genes and poses a grave threat if it spreads widely in the community.^35,36^

One major limitation of this study was that we could only generate short-read genome sequences. The use of long-read sequencing would have enabled determination of the genetic environment harbouring the NDM-5 gene within each bacterial species to identify if it was carried on a species-specific plasmid or within the chromosome. Another limitation was the cross-sectional design, which limited our ability to track the longitudinal differences in transmission dynamics post hospitalization within households. In addition, further studies from the environment and food sources are required to identify the reservoirs in the community and mechanisms of dissemination of antimicrobial resistant bacteria to the wider population. Recent studies have shown that MDR healthcare-associated bacteria share mobile genetic elements (MGEs) across large phylogenetic distances.^40–42^ Identifying the dynamics of MGE transmission in community settings can explain important epidemiological links that remain unsolved.^43,44^

### Conclusion

Our study shows that the prevalence of ESBL *E. coli* and *K. pneumoniae*/*K. quasipneumoniae* in Cambodia has not only increased considerably compared with previous reports but is also diverse genotypically. ESBL genes, specifically *bla*_CTX-M_, are now found in almost all *E. coli* commensals, indicating that they are continuously propagated in the community through various currently unknown channels. Another startling finding was the presence of *bla*_NDM-5_ in both *E. coli* and *K. pneumoniae* from healthy individuals. If carbapenem resistance prevalence increases at a similar rate as was seen for ESBL, then untreatable Enterobacterales will be commonplace in Cambodia in the near future. Effective community-level interventions are required urgently.

## Supplementary Material

dlad067_Supplementary_Data

## Supplementary data

Figures S1 and S2 and Tables S1 to S9 are available as Supplementary data at *JAC-AMR* Online.
