# Supplementary material for: Whole genome sequencing of multidrug resistant Enterobacterales identified in children and their household members within Siem Reap, Cambodia
Source: JAC Antimicrob Resist. 2023 Jun 14;5(3):dlad067. doi: 10.1093/jacamr/dlad067 (PMC10265595; doi:10.1093/jacamr/dlad067)
Supplement: dlad067_Supplementary_Data [file dlad067_supplementary_data.docx]

**Table S1: Sequence type distribution of *E. coli* isolates obtained in this study (n ≥ 3)**

| **Sequence type** | **Hospital households** | **Community households** | **Total** | **p-value** |
| --- | --- | --- | --- | --- |
| ST10/10^#^ | 14 | 6 | 20 | 0.35 |
| ST131 | 10 | 5 | 15 | 0.55 |
| ST1193 | 9 | 5 | 14 |  |
| ST38 | 4 | 9 | 13 | 0.04 |
| ST648 | 7 | 1 | 8 | 0.14 |
| ST410 | 8 | 1 | 9 | 0.08 |
| ST48 | 4 | 3 | 7 | - |
| ST156 | 2 | 5 | 7 | - |
| ST394/394^#^ | 3 | 4 | 7 | - |
| ST69 | 4 | 2 | 6 | - |
| ST215 | 1 | 5 | 6 | - |
| ST421 | 2 | 2 | 4 | - |
| ST1722 | 3 | 1 | 4 | - |
| ST93 | 2 | 2 | 4 | - |
| ST224 | 2 | 2 | 4 | - |
| ST226 | 1 | 3 | 4 | - |
| ST278 | 1 | 2 | 3 | - |
| ST354 | 3 | 0 | 3 | - |
| ST746 | 2 | 1 | 3 | - |
| ST196 | 3 | 0 | 3 | - |
| ST617 | 2 | 1 | 3 | - |
| ST206 | 1 | 2 | 3 | - |
| ST29 | 2 | 1 | 3 | - |
| ST1312 | 1 | 2 | 3 | - |
| ST757 | 3 | 0 | 3 | - |
| ST162 | 2 | 1 | 3 | - |
| ST155 | 1 | 2 | 3 | - |

^#^-single locus variant (reported as 1LV in Kleborate)

**Table S2:** **Sequence type distribution of *K. pneumoniae* and *K. quasipneumoniae* isolates obtained in this study (n ≥ 2)**

| ***K. pneumoniae*** | **Number** |  | ***K. quasipneumoniae*** | **Number** |
| --- | --- | --- | --- | --- |
| ST101 | 9 |  | ST1308 | 8 |
| ST37 | 4 |  | ST1584 | 4 |
| ST1741 | 4 |  | ST841 | 4 |
| ST967 | 4 |  | ST1795/1795^$^ | 2 |
| ST307 | 3 |  | ST1770 | 2 |
| ST76 | 3 |  | ST1504/1504^$^ | 2 |
| ST3113 | 3 |  | ST1699 | 2 |
| ST1059/1059^#^ | 3 |  | ST841/841^#^ | 2 |
| ST17 | 3 |  |  |  |
| ST1207 | 2 |  |  |  |
| ST37/37^#^ | 2 |  |  |  |
| ST327 | 2 |  |  |  |
| ST15 | 2 |  |  |  |
| ST152 | 2 |  |  |  |
| ST534/534^#^ | 2 |  |  |  |
| ST774 | 2 |  |  |  |
| ST2670 | 2 |  |  |  |
| ST1047 | 2 |  |  |  |

^#^-single locus variant (reported as 1LV in Kleborate)

^$^-double locus variant (reported as 2LV in Kleborate)

**Table S3. *E. coli* distribution of major ST and ESBL genes**

| **ST** | **CTX-M-15** | **CTX-M-55** | **CTX-M-27** | **CTX-M-14** | **CTX-M-24** | **CMY-42** |
| --- | --- | --- | --- | --- | --- | --- |
| **ST10** | 7 | 8 | 1 | 1 |  |  |
| **ST131** | 5 |  | 10 |  |  |  |
| **ST1193** | 2 | 1 | 11 |  |  |  |
| **ST38** | 5 |  | 4 | 3 | 1 |  |
| **ST410** | 4 | 2 | 2 |  |  | 2 |
| **ST648** | 4 |  | 1 | 3 |  | 1 |
| **ST156** | 3 | 4 |  |  |  |  |
| **ST48** |  | 5 |  | 1 |  |  |
| **ST394** | 3 | 1 | 1 | 1 |  |  |
| **ST69** | 1 | 1 | 3 | 1 |  |  |
| **ST215** | 4 | 1 |  |  |  |  |

**Table S4. *Klebsiella spp.* distribution of major ST and ESBL genes**

| ***K. pneumoniae*** | | | | |
| --- | --- | --- | --- | --- |
| **ST** | **CTX-M-15** | **CTX-M-55** | **CTX-M-27** | **CTX-M-3** |
| **ST101** | 8 | 1 |  |  |
| **ST967** | 3 |  |  | 1 |
| **ST37** | 3 |  | 1 |  |
| **ST1741** | 4 |  |  |  |
| ***K. quasipneumonaie*** | | | | |
| **ST** | **CTX-M-15** | **CTX-M-55** | **CTX-M-14** | **CTX-M-27** |
| **ST1308** | 7 |  |  |  |
| **ST1584** | 2 |  |  | 1 |
| **ST841** |  | 1 | 3 |  |

**Table S5: Matching isolate pairs in the community: *E. coli***

| **Sr no.** | **Participant ID** | ***E. coli* MLST** | **Pairwise**  **SNP distance** | **ESBL genes** | **Same household** |
| --- | --- | --- | --- | --- | --- |
| 1 | 004-03;  141-01 | ST2325 | 10 | CTX-M-55 | No |
| 2 | 005-03;  105-03 | ST5215 | 3 | CTX-M-55 | No |
| 3 | 005-04*;  144-01 | ST656 | 8 | CTX-M-55 | No |
| 4 | 006-03;  109-02 | ST10 | 10 | CTX-M-15 | No |
| 5 | 007-02; 110-03 | ST1193 | 9 | CTX-M-27 | No |
|  | 007-02; 110-05 | ST1193 | 6 | CTX-M-27 | No |
|  | 110-03; 110-05 | ST1193 | 4 | CTX-M-27 | Yes |
| 6 | 008-02;  008-04 | ST540 | 10 | CTX-M-15 | Yes |
| 7 | 012-01; 040-05 | ST761 | 3 | CTX-M-15 | No |
| 8 | 012-04;  020-03 | ST131 | 7 | CTX-M-27 | No |
|  | 012-05;  020-03 | ST131 | 7 | CTX-M-27 | No |
|  | 012-04;  012-05 | ST131 | 7 | CTX-M-27 | Yes |
| 9 | 017-02;  017-08 | ST7219 | 8 | CTX-M-27 | Yes |
| 10 | 019-01;  019-07 | ST10 | 5 | CTX-M-55 | Yes |
| 11 | 023-02;  129-01 | ST162 | 8 | CTX-M-15 | No |
| 12 | 027-02;  027-06 | ST542 | 7 | CTX-M-55 | Yes |
| 13 | 035-01; 037-02* | ST2539 | 7 | CTX-M-55 | No |
| 14 | 036-02; 036-03 | ST421 | 3 | CTX-M-27 | Yes |
| 15 | 041-03*; 044-03 | ST1193 | 8 | CTX-M-27 | No |
| 16 | 110-01;  110-04 | ST215 | 3 | CTX-M-15 | Yes |
|  | 110-01;  110-02 | ST215 | 4 | CTX-M-15 | Yes |
|  | 110-02;  110-04 | ST215 | 6 | CTX-M-15 | Yes |
|  | 110-01;  137-02 | ST215 | 10 | CTX-M-15 | No |
|  | 110-04;  137-02 | ST215 | 8 | CTX-M-15 | No |
| 17 | 126-01;  126-04 | ST421 | 4 | CTX-M-27 | Yes |
| 18 | 134-04;  134-05 | ST10 | 6 | CTX-M-15 | Yes |

*- history of recent hospitalization (14-28 days before sample collection)

**Table S6: Matching isolate pairs in the community: *K. pneumoniae***

| **Sr no.** | **Participa-nt ID** | ***K. pneumoniae***  **MLST** | **Pairwise**  **SNP distance** | **ESBL genes** | **Same household** |
| --- | --- | --- | --- | --- | --- |
| 1 | 002-07;  002-08 | ST967 | 1 | CTX-M-15 | Yes |
| 2 | 003-01; 003-02 | ST534-1LV | 8 | CTX-M-55 | Yes |
| 3 | 003-03*; 010-02 | ST1059-1LV | 6 | CTX-M-55 | No |
|  | 003-03; 140-01 | ST1059-1LV | 10 | CTX-M-55 | No |
|  | 010-02;  140-01 | ST1059-1LV | 5 | CTX-M-55 | No |
| 4 | 005-03; 105-01 | ST101 | 2 | CTX-M-15 | No |
|  | 005-03;  105-03 | ST101 | 6 | CTX-M-15 | No |
|  | 105-01; 105-03 | ST101 | 6 | CTX-M-15 | Yes |
| 5 | 006-02*;  106-01 | ST152 | 5 | CTX-M-14 | No |
| 6 | 006-04; 101-01 | ST1207 | 6 | CTX-M-15 | No |
| 7 | 027-05*; 044-02 | ST774 | 4 | CTX-M-15 | No |
| 8 | 118-01; 118-03 | ST37-1LV | 4 | CTX-M-24 | Yes |
| 9 | 134-04; 134-05 | ST76 | 10 | CTX-M-15 | Yes |
| 10 | 143-03; 143-04 | ST1047 | 5 | CTX-M-9 | Yes |
| 11 | 144-03; 144-04 | ST101 | 4 | CTX-M-15 | Yes |

*- history of recent hospitalization (14-28 days before stool sample collection)

**Table S7: *E.coli* phenotypic susceptibility and Antibiotic resistant genes (n = 276)**

| **Drug** | **Phenotype** | **Genes (WGS)** | **Variation** | **Total** |
| --- | --- | --- | --- | --- |
| Ciprofloxacin  S: 14  NS: 252 | S | - | NA | 14 |
|  | S | qnrS1 |  | 9 |
|  | S | qnrB4 |  | 1 |
|  | NS | - | NA | 98 |
|  | NS | qnrS1 | qnrS1* | 132 |
|  | NS | qnrS2 |  | 2 |
|  | NS | qepA2* |  | 8 |
|  | NS | qepA2*;qnrS1 |  | 1 |
|  | NS | qnrB19^ |  | 1 |
|  | NS | qnrB4 |  | 7 |
|  | NS | qnrB4;qnrS1 |  | 2 |
|  | NS | qnrS4 |  | 1 |
| Co-Trimoxazole (Sulmethoxazole genes)  S: 85  NS: 191 | S | - |  | 69 |
|  | S | sul1 |  | 2 |
|  | S | sul2 | sul2*  sul2^ | 8 |
|  | S | sul3 | sul3^ | 6 |
|  | NS | - |  | 9 |
|  | NS | sul1 | sul1* | 40 |
|  | NS | sul1;sul2 | sul1;sul2*  sul1*;sul2*  sul1;sul2^  sul1?;sul2*  sul1^;sul2 | 60 |
|  | NS | sul2 | sul2*  sul2^ | 52 |
|  | NS | sul3 | sul3^ | 18 |
|  | NS | sul1;sul3 |  | 1 |
|  | NS | sul2;sul3 | sul2;sul3^ | 10 |
|  | NS | sul1;sul2;sul3 |  | 1 |
| Co-Trimoxazole (Trimethoprim)  S: 85  NS: 191 | S | - |  | 83 |
|  | S | dfrA12 |  | 2 |
|  | NS | - |  | 3 |
|  | NS | dfrA17 |  | 74 |
|  | NS | dfrA14.v2* | dfrA14.v2*? | 54 |
|  | NS | dfrA12 | dfrA12^ | 30 |
|  | NS | dfrA1.v2 |  | 8 |
|  | NS | dfrA1.v2;dfrA17 |  | 3 |
|  | NS | dfrA5 |  | 7 |
|  | NS | dfrA7 |  | 2 |
|  | NS | dfrA8 |  | 1 |
|  | NS | dfrA12;dfrA7 |  | 1 |
|  | NS | dfrA12;dfrA17 |  | 3 |
|  | NS | dfrA12;dfrA14.v2* |  | 1 |
|  | NS | dfrA1.v2;dfrA14.v2* |  | 2 |
|  | NS | dfrA1.v2;dfrA12 |  | 1 |
|  | NS | dfrA1.v1;dfrA12 |  | 1 |
| Chloramphenicol  S: 189  NS: 87 | S | - |  | 177 |
|  | S | catA1^ |  | 4 |
|  | S | catB4.v1? |  | 7 |
|  | S | cmlA5 |  | 1 |
|  | NS | - |  | 5 |
|  | NS | floR.v1 | floR.v1* | 22 |
|  | NS | floR.v2* |  | 9 |
|  | NS | catII.2* |  | 17 |
|  | NS | catA1* | catA1^ | 16 |
|  | NS | catB3.v2;floR.v1* |  | 1 |
|  | NS | catB3.v2;floR.v2* |  | 1 |
|  | NS | catB4.v1? |  | 1 |
|  | NS | catB4.v1?;catA1^ |  | 2 |
|  | NS | catB4.v1?;floR.v1* |  | 1 |
|  | NS | catII.2*;cmlA1* |  | 1 |
|  | NS | catII.2*;floR.v1 | catII.2*;floR.v1* | 3 |
|  | NS | cmlA1* |  | 5 |
|  | NS | cmlA1*;floR.v2* |  | 2 |
|  | NS | cmlA5;floR.v1* |  | 1 |
| Gentamicin  S: 194  NS: 82 | S | - |  | 75 |
|  | S | aac(3)-IId^;aadA2^ |  | 2 |
|  | S | aac(6')-Ib-cr.v2;aadA2^;aadA5 |  | 1 |
|  | S | aac(6')-Ib-cr.v2;aadA2^;strA.v1^;strB.v1 |  | 1 |
|  | S | aac(6')-Ib-cr.v2;aadA5 |  | 2 |
|  | S | aadA;aadA2^ |  | 1 |
|  | S | aadA* | aadA^ | 2 |
|  | S | aadA*;aph3-Ia.v1^ |  | 2 |
|  | S | aadA*;strA.v1^;strB.v1 |  | 1 |
|  | S | aadA^;strA.v1;strB.v1 |  | 1 |
|  | S | aadA1.v1^ |  | 10 |
|  | S | aadA2^ |  | 4 |
|  | S | aadA2^;aadA^ |  | 1 |
|  | S | aadA2^;aadA^;ant(2'')-Ia;aph3-Ia.v1^ |  | 1 |
|  | S | aadA2^;strA.v1*;strB.v1 | aadA2^;strA.v1^;strB.v1* | 2 |
|  | S | aadA2^;strA.v1^ |  | 1 |
|  | S | aadA5 | aadA5* | 15 |
|  | S | aadA5;strA.v1*;strB.v1* | aadA5;strA.v1^;strB.v1 | 26 |
|  | S | aph(3')-Ia* |  | 2 |
|  | S | aph3-Ia.v1^;strA.v1^;strB.v1 |  | 2 |
|  | S | strA.v1*;strB.v1* | strA.v1;strB.v1  strA.v1^;strB.v1*  strA.v1^;strB.v1 | 38 |
|  | S | strA.v1^ | strA.v1* | 3 |
|  | S | strB.v1 |  | 1 |
|  | NS | - |  | 1 |
|  | NS | aac(3)-IIa.v1^ |  | 3 |
|  | NS | aac(3)-IIa.v1^;aac(6')-Ib-cr.v2 |  | 1 |
|  | NS | aac(3)-IIa.v1^;aac(6')-Ib-cr.v2;aadA5 |  | 1 |
|  | NS | aac(3)-IIa.v1^;aac(6')-Ib-cr.v2;aadA5;strA.v1^;strB.v1 | aac(3)-IIa.v1^;aac(6')-Ib-cr.v2;aadA5;strA.v1^;strB.v1* | 2 |
|  | NS | aac(3)-IIa.v1^;aadA^;strA.v1*;strB.v1* |  | 1 |
|  | NS | aac(3)-IIa.v1^;aph3-Ia.v1^ |  | 4 |
|  | NS | aac(3)-IIa.v1^;aph3-Ia.v1^;strA.v1*;strB.v1* | aac(3)-IId*?;aph(3')-Ia*;strA.v1*;strB.v1* | 3 |
|  | NS | aac(3)-IId^ |  | 4 |
|  | NS | aac(3)-IId^;aac(3)-VIa*;aadA2^;strA.v1;strB.v1 |  | 1 |
|  | NS | aac(3)-IId^;aac(6')-Ib-cr.v2 |  | 1 |
|  | NS | aac(3)-IId^;aac(6')-Ib-cr.v2;aadA2^;aadA5;strA.v1^;strB.v1 |  | 1 |
|  | NS | aac(3)-IId^;aac(6')-Ib-cr.v2;aadA22;aph(3')-Ia*;strB.v1 | aac(3)-IId^;aac(6')-Ib-cr.v2^;aadA22;aph(3')-Ia*;strB.v1 | 2 |
|  | NS | aac(3)-IId^;aadA*;aadA2^;sat-2;strA.v1;strB.v1 |  | 1 |
|  | NS | aac(3)-IId^;aadA*;aph3-Ia.v1^;strA.v1*;strB.v1* | aac(3)-IId^;aadA^;aph3-Ia.v1^;strA.v1*;strB.v1* | 2 |
|  | NS | aac(3)-IId^;aadA2 |  | 1 |
|  | NS | aac(3)-IId^;aadA2;aadA? | ac(3)-IId^;aadA2;aadA^ | 2 |
|  | NS | aac(3)-IId^;aadA2;aadA^;aph3-Ia.v1^;strA.v1;strB.v1 |  | 1 |
|  | NS | aac(3)-IId^;aadA2;strA.v1;strB.v1 |  | 1 |
|  | NS | aac(3)-IId^;aadA2^ |  | 2 |
|  | NS | aac(3)-IId^;aadA2^;aadA^ |  | 1 |
|  | NS | aac(3)-IId^;aadA2^;aadA^;aph3-Ia.v1^;strA.v1;strB.v1 | aac(3)-IId^;aadA2^;aadA^;aph3-Ia.v1^;strA.v1^;strB.v1* | 2 |
|  | NS | aac(3)-IId^;aadA2^;strA.v1;strB.v1 | aac(3)-IId^;aadA2^;strA.v1*;strB.v1*  aac(3)-IId^;aadA2^;strA.v1^;strB.v1 | 4 |
|  | NS | aac(3)-IId^;aadA22;aph(3')-Ia*;strA.v1*;strB.v1* |  | 1 |
|  | NS | aac(3)-IId^;aadA22* | aac(3)-IId^;aadA22^ | 4 |
|  | NS | aac(3)-IId^;aadA22*;aph3-Ia.v1^ |  | 3 |
|  | NS | aac(3)-IId^;aadA22^;strA.v1^;strB.v1 |  | 1 |
|  | NS | aac(3)-IId^;aadA5 |  | 3 |
|  | NS | aac(3)-IId^;aadA5;aph(3')-IIa |  | 1 |
|  | NS | aac(3)-IId^;aadA5;strA.v1;strB.v1 | aac(3)-IId^;aadA5;strA.v1;strB.v1^  aac(3)-IId^;aadA5;strA.v1^;strB.v1  aac(3)-IId^;aadA5;strA.v1^;strB.v1* | 16 |
|  | NS | aac(3)-IId^;aph3-Ia.v1^ |  | 1 |
|  | NS | aac(3)-IId^;strA.v1;strB.v1 | aac(3)-IId^;strA.v1*;strB.v1*  aac(3)-IId^;strA.v1*;strB.v1*?  aac(3)-IId^;strA.v1^;strB.v1  aac(3)-IId^;strA.v1^;strB.v1* | 9 |
|  | NS | aac(3)-VIa*;aadA8b* |  | 1 |

Phenotype: Non-susceptibility (NS) (intermediate and resistant)

Susceptibility (S)

*no precise nucleotide or amino acid match is found, but the closest nucleotide match

^exact match to protein sequence but with one or more nucleotide differences

**Table S8: *K. pneumoniae* phenotypic susceptibility and Antibiotic resistant genes (n=89)**

| **Drug** | **Phenotype** | **Genes (WGS)** | **Variation** | **Total** |
| --- | --- | --- | --- | --- |
| Ciprofloxacin  (Flq acquired)  S: 6  NS: 83 | S | - |  | 6 |
|  | NS | qnrS1 |  | 58 |
|  | NS | qnrB4 |  | 6 |
|  | NS | qnrB6^ |  | 6 |
|  | NS | qnrB6^;qnrS1 |  | 1 |
|  | NS | qnrB1.v2^ |  | 10 |
|  | NS | qnrB2.v2^;qnrS1 |  | 1 |
|  | NS | qnrA1 |  | 1 |
| Co-trimoxazole  (Sulmethoxazole)  S: 29  NS: 60 | S | - |  | 23 |
|  | S | sul1 |  | 4 |
|  | S | sul1;sul2 |  | 1 |
|  | S | sul2 | sul2* | 8 |
|  | NS | - |  | 1 |
|  | NS | sul1 | sul1? | 13 |
|  | NS | sul1;sul1 | sul1;sul1*  sul1;sul1^ | 6 |
|  | NS | sul1;sul1;sul2 | sul1;sul1;sul2* | 3 |
|  | NS | sul1;sul2 | sul1;sul2* | 6 |
|  | NS | sul2 | sul2* | 28 |
|  | NS | sul3 |  | 3 |
| Co-trimoxazole  (Trimethoprim)  S: 29  NS: 60 | S | - |  | 27 |
|  | S | dfrA12 |  | 1 |
|  | S | dfrA14.v2* |  | 1 |
|  | NS | - |  | 2 |
|  | NS | dfrA1.v1 |  | 6 |
|  | NS | dfrA1.v1;dfrA15.v2 |  | 1 |
|  | NS | dfrA1.v2 |  | 5 |
|  | NS | dfrA1.v2;dfrA27 |  | 1 |
|  | NS | dfrA12 |  | 6 |
|  | NS | dfrA14.v2* |  | 27 |
|  | NS | dfrA14.v2*;dfrA5 |  | 1 |
|  | NS | dfrA27 |  | 11 |
| Chloramphenicol  S: 66  NS: 23 | S | - |  | 56 |
|  | S | catB3.v2 |  | 1 |
|  | S | catB4.v1? |  | 8 |
|  | S | floR.v1* |  | 1 |
|  | NS | - |  | 2 |
|  | NS | catA1^ |  | 4 |
|  | NS | catB4.v1? |  | 1 |
|  | NS | catII.2* |  | 3 |
|  | NS | cmlA1* |  | 1 |
|  | NS | floR.v1 | floR.v1* | 9 |
|  | NS | floR.v2* |  | 3 |
| Gentamicin  S: 70  NS: 19 | S | - |  | 27 |
|  | S | aac(6')-Ib-cr.v2;aadA;aadA16* |  | 1 |
|  | S | aac(6')-Ib-cr.v2;aadA16* |  | 5 |
|  | S | aac(6')-Ib-cr.v2;aadA16*;aph3-Ia.v1 |  | 1 |
|  | S | aac(6')-Ib-cr.v2;aadA16*;strA.v1*;strB.v1* |  | 1 |
|  | S | aac(6')-Ib-cr.v2^ |  | 1 |
|  | S | aadA | aadA^ | 4 |
|  | S | aadA2^ |  | 2 |
|  | S | aadA2^;aadA^;aph(3')-IIa;strA.v1;strB.v1 |  | 1 |
|  | S | aadA2^;aph3-Ia.v1^;strA.v1*;strB.v1* |  | 1 |
|  | S | aadA2^;strA.v1 |  | 1 |
|  | S | aadA5 |  | 1 |
|  | S | aph(3'')-Ib*;strB.v1^ |  | 1 |
|  | S | strA.v1;strB.v1 | strA.v1*;strB.v1*  strA.v1^;strB.v1  strA.v1^;strB.v1^ | 23 |
|  | NS | aac(3)-IIa.v1^;aac(6')-Ib-cr.v2;aadA2^ |  | 1 |
|  | NS | aac(3)-IIa.v1^;aac(6')-Ib-cr.v2;strA.v1^;strB.v1 |  | 8 |
|  | NS | aac(3)-IId^;aac(6')-Ib-cr.v2;aadA16*;aph3-Ia.v1;strA.v1^;strB.v1 |  | 2 |
|  | NS | aac(3)-IId^;aac(6')-Ib-cr.v2;aadA16*;strA.v1*;strB.v1* |  | 1 |
|  | NS | aac(3)-IId^;aadA2^;aph3-Ia.v1^ |  | 1 |
|  | NS | aac(3)-IId^;aadA22^;strB.v1 |  | 2 |
|  | NS | aac(3)-IId^;aadA5;strA.v1;strB.v1 |  | 1 |
|  | NS | aac(3)-IId^;strA.v1^;strB.v1 |  | 2 |
|  | NS | aac(6')-Ib-cr.v2;aadA16* |  | 1 |

ARGs: Antibiotic resistant genes

Phenotype: Non-susceptibility (NS) (intermediate and resistant)

Susceptibility (S)

*no precise nucleotide or amino acid match is found, but the closest nucleotide match

^exact match to protein sequence but with one or more nucleotide differences

**Table S9: *K. quasipneumoniae* phenotypic susceptibility and Antibiotic resistant genes (n=40)**

| **Drug** | **Phenotype** | **Genes (WGS)** | **Variation** | **Total** |
| --- | --- | --- | --- | --- |
| Ciprofloxacin  (Flq acquired)  S: 3  NS: 37 | S | - |  | 3 |
|  | NS | - |  | 1 |
|  | NS | qnrS1 |  | 26 |
|  | NS | qnrB4 |  | 4 |
|  | NS | qnrB1.v2^ |  | 4 |
|  | NS | qnrB2.v2^;qnrS1 |  | 1 |
|  | NS | qnrA1^ |  | 1 |
| Co-trimoxazole  (Sulmethoxazole)  S: 11  NS: 29 | S | - |  | 7 |
|  | S | sul2 | sul2* | 4 |
|  | NS | - |  | 3 |
|  | NS | sul1 | sul1? | 5 |
|  | NS | sul1;sul1 |  | 1 |
|  | NS | sul1*;sul1*;sul2 |  | 1 |
|  | NS | sul1;sul2 |  | 3 |
|  | NS | sul2 | sul2* | 16 |
| Co-trimoxazole  (Trimethoprim)  S: 11  NS: 29 | S | - |  | 11 |
|  | NS | dfrA1.v1 |  | 3 |
|  | NS | dfrA1.v2 |  | 2 |
|  | NS | dfrA12 |  | 2 |
|  | NS | dfrA14.v2* |  | 16 |
|  | NS | dfrA5 |  | 2 |
|  | NS | dfrA15.v2 |  | 2 |
|  | NS | dfrA17 |  | 1 |
|  | NS | dfrA27 |  | 1 |
| Chloramphenicol  S: 35  NS: 5 | S | - |  | 31 |
|  | S | catB4.v1? |  | 3 |
|  | S | floR.v1* |  | 1 |
|  | NS | catA1* |  | 1 |
|  | NS | catB4.v1?;catA1* |  | 1 |
|  | NS | floR.v1 | floR.v1* | 3 |
| Gentamicin  S: 35  NS: 5 | S | - |  | 15 |
|  | S | aac(3)-IId^;strA.v1;strB.v1 |  | 1 |
|  | S | aac(6')-Ib-cr.v2;strA.v1^;strB.v1 |  | 2 |
|  | S | aadA^;strA.v1;strB.v1 |  | 2 |
|  | S | aadA2^;aph3-Ia.v1^ |  | 1 |
|  |  | aadA2^;strA.v1*;strB.v1* |  | 1 |
|  | S | aadA5 |  | 2 |
|  | S | strA.v1;strB.v1 | strA.v1*;strB.v1*  strA.v1^;strB.v1  strA.v1^;strB.v1^ | 11 |
|  | NS | aac(3)-IIa.v1^;aac(6')-Ib-cr.v2;strA.v1^;strB.v1 |  | 2 |
|  | NS | aac(3)-IId^ |  | 1 |
|  | NS | aac(3)-IId^;aadA5;strA.v1;strB.v1 |  | 1 |
|  | NS | aac(6')-Ib-cr.v2;aadA16*;strA.v1*;strB.v1* |  | 1 |

ARGs: Antibiotic resistant genes

Phenotype: Non-susceptibility (NS) (intermediate and resistant)

Susceptibility (S)

*no precise nucleotide or amino acid match is found, but the closest nucleotide match

^exact match to protein sequence but with one or more nucleotide differences

**Figure S1. Genetic structure of the contigs harbouring CTX-M-27 in *E. coli***


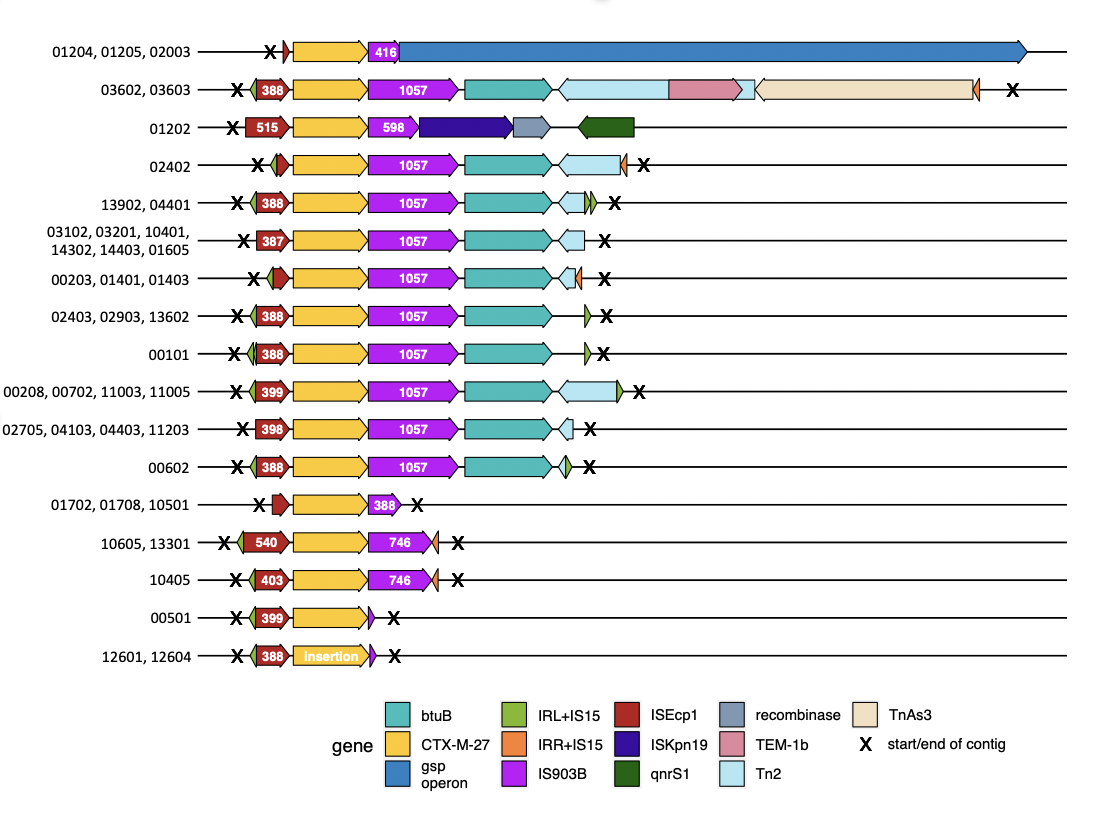


Footnotes: IRL = left inverted repeat region

IRR = right inverted repeat region

Number in arrow indicates gene length in base pair

**Figure S2: Genetic structure of the contigs harbouring CTX-M-27 in *K. pneumoniae***

Footnotes: IRL = left inverted repeat region

IRR = right inverted repeat region

CDS = coding sequence

∆ = truncated gene

Number in arrow indicates gene length in base pair
